# Transcription Factor *GmWRKY46* Enhanced Phosphate Starvation Tolerance and Root Development in Transgenic Plants

**DOI:** 10.3389/fpls.2021.700651

**Published:** 2021-09-14

**Authors:** Cheng Li, Kangning Li, Xinyi Liu, Hui Ruan, Mingming Zheng, Zhijie Yu, Junyi Gai, Shouping Yang

**Affiliations:** ^1^Soybean Research Institute, National Center for Soybean Improvement, Key Laboratory of Biology and Genetic Improvement of Soybean (General, Ministry of Agriculture), Jiangsu Collaborative Innovation Center for Modern Crop Production, Nanjing Agricultural University, Nanjing, China; ^2^State Key Laboratory of Crop Genetics and Germplasm Enhancement, Nanjing Agricultural University, Nanjing, China; ^3^MOA Key Laboratory of Plant Nutrition and Fertilization in Lower-Middle Reaches of the Yangtze River, Nanjing Agricultural University, Nanjing, China

**Keywords:** soybean (*Glycine max*), *GmWRKY46*, phosphate starvation, root development, RNA-Seq, *AED1*

## Abstract

Phosphorus (P) is one of the essential macronutrients, whose deficiency limits the growth and development of plants. In this study, we investigated the possible role of *GmWRKY46* in the phosphate (Pi) starvation stress tolerance of soybean. GmWRKY46 belonged to the group III subfamily of the WRKY transcription factor family, which was localized in the nucleus and had transcriptional activator activity. *GmWRKY46* could be strongly induced by Pi starvation, especially in soybean roots. Overexpression of *GmWRKY46* significantly enhanced tolerance to Pi starvation and lateral root development in transgenic *Arabidopsis*. RNA-seq analysis showed that overexpression of *GmWRKY46* led to change in many genes related to energy metabolisms, stress responses, and plant hormone signal transduction in transgenic *Arabidopsis*. Among these differential expression genes, we found that overexpression of *AtAED1* alone could enhance the tolerance of transgenic *Arabidopsis* to Pi starvation. Y1H and ChIP-qPCR analyses showed that GmWRKY46 could directly bind to the W-box motif of the *AtAED1* promoter *in vitro* and *in vivo*. Furthermore, results from intact soybean composite plants with *GmWRKY46* overexpression showed that *GmWRKY46* was involved in hairy roots development and subsequently affected plant growth and Pi uptake. These results provide a basis for the molecular genetic breeding of soybean tolerant to Pi starvation.

## Introduction

Phosphorus (P) is one of the most essential mineral nutrients required for the growth and development of plants and is a constituent of key molecules such as ATP, nucleic acids, and phospholipids (Chiou and Lin, 2011). It plays a crucial role in energy generation, photosynthesis, glycolysis, respiration, protein activation, and stability. Plants meet their P requirement only by taking up inorganic phosphate (Pi) from the soil. Although P is abundant in many soils, it is rarely present in the form of Pi that can be used by plants, so crop yield on 30–40% of the arable land of the world is limited by P availability (Vance et al., 2003). At present, intensive application of chemical fertilizers containing Pi has become a standard agricultural practice to ensure crop productivity (Chiou and Lin, 2011). However, the excess Pi application is not a perfect solution. On the one hand, the excess dissolution of Pi pollutes water sources (MacDonald et al., 2011). On the other hand, most of the annually fertilized Pi is fixed in the soil in organic forms which are unavailable to plants in the absence of mineralization (Raghothama, 1999). To cope with Pi deficiency, plants themselves have evolved several ways to optimize Pi acquisition from soil (Cong et al., 2020). For example, stimulating lateral root and hairy root growth leads to profound changes in root structure, thereby increasing the Pi absorption surface of the root system (Williamson et al., 2001; Ticconi and Abel, 2004; Svistoonoff et al., 2007), releasing organic acids and phosphatases to release Pi by dissolving organic P (Jones, 1998; Hinsinger, 2001), and getting more Pi by symbiosis with mycorrhizal fungi (Javot et al., 2007). In-depth research on the mechanism of how plants resist Pi starvation can provide effective references to future plant production.

Phosphate starvation responses in plants are tightly regulated by an elaborate signaling network that comprises multiple components that are not fully understood. In this network, transcription factors (TFs) act as significant coordinators to transfer stress signals and to orchestrate the expression of their target genes (Huang et al., 2018). They play crucial roles in protecting against stress-associated damage by modulating the expression level of downstream target genes (Vigeolas et al., 2008). In recent years, researchers have used molecular biology methods to find that many families of TFs play important roles in plant signaling responses to low Pi including WRKY, MYB, AP2/ERF, and bHLH family members (Baek et al., 2013; Dai et al., 2015; Yang et al., 2016a; Chen et al., 2018). The WRKY TF family is one of the largest TF families in plants, which, based on the highly conserved WRKY domain, has 72 members in *Arabidopsis* (*Arabidopsis thaliana*) and 182 members in soybean (*Glycine max*) (Eulgem and Somssich, 2007; Bencke-Malato et al., 2014). The WRKY domain is a conserved DNA-binding region that includes highly conserved WRKYGQK peptide sequences and zinc finger motifs which can be either C_2_H_2_-type (Cx_4−5_Cx_22−23_HxH) or C_2_HC-type (Cx_7_Cx_23_HxC) (Rushton et al., 2010). Members of the WRKY family have been found to contain at least one such domain. The WRKY domain generally binds to the promoter region of target genes containing the W-box(es) sequence (C/TTGACT/C), although alternative binding sites have been identified (Pandey and Somssich, 2009). WRKY TFs are involved in responses to biotic and abiotic stresses, and developmental processes (Eulgem and Somssich, 2007; Pandey and Somssich, 2009). It has been found that some WRKY TFs are particularly sensitive to Pi starvation in plants. For example, *AtWRKY45* overexpression in *Arabidopsis* increased the Pi content and uptake, while RNA interference suppression of *AtWRKY45* decreased the Pi content and uptake (Wang et al., 2014). Overexpression of *OsWRKY74* significantly enhanced tolerance to Pi starvation in rice (*Oryza sativa*), whereas transgenic lines with downregulation of *OsWRKY74* were sensitive to Pi starvation (Dai et al., 2015).

Aspartic proteinases (APs) are an important class of proteolytic enzymes, which occur in a wide variety of plants and participate in many important physiological processes (Mutlu and Gal, 1999). With the improvement of genome sequencing technology, more and more plant APs genes have been detected. About 69 genes are encoding APs in the *Arabidopsis* genome, 96 AP genes in the rice genome, and 50 AP genes in the grape (*Vitis vinifera*) genome (Takahashi et al., 2008; Chen et al., 2009; Guo et al., 2013). APs play important roles in the whole growth and development of plants, especially under stress response, sexual reproduction, aging, programmed cell death as well as processing and degradation of proteins. To date, it has been proved that some plant APs play an important role in abiotic stress. *ASPG1* (ASPARTIC PROTEASE IN GUARD CELL 1) in *Arabidopsis* is a typical example of plant APs participating in abiotic stress, which is involved in the ABA signaling pathway and mediates the response to plant drought stress by regulating the balance of ROS levels in cells (Yao et al., 2012). In addition, aspartic proteases responding to abiotic stress have also been identified in common bean (*Phaseolus vulgaris*), pineapple (*Ananas comosus*), buckwheat (*Fagopyrum esculentum Moench*.), and other species (Contouransel et al., 2010; Timotijevic et al., 2010; Raimbault et al., 2013). However, as far as we are aware, no studies have found evidence that APs are involved in Pi starvation.

Soybean (*Glycine max*) is one of the most important crops for oil and protein production, and its yield is severely affected by various environmental conditions. P deficiency is more likely to be a limiting factor for soybean yield because of the high demand for P in the nodule responsible for nitrogen fixation (Vance, 2001; Song et al., 2014). So far, botanists have identified hundreds of genes related to how plants adapt to Pi starvation, but most research has focused on model plants, with soybean relatively lagging (Zhang et al., 2014). Here, we investigated the function of *GmWRKY46* and characterized it as a regulator of Pi starvation responses. We found that *GmWRKY46* enhanced tolerance to Pi starvation through improving root development and direct interaction with the *AtAED1* promoter in transgenic *Arabidopsis*. Besides, we further found that overexpressing *GmWRKY46* in soybean transgenic hairy roots could enhance Pi starvation tolerance and root development. This study laid a foundation for the improvement of the low P tolerance of soybean in the future.

## Materials and Methods

### Phylogenetic and Gene Structure Analysis

Plant Comparative Genomics portal (Department of Energy, Joint Genome Institute, https://phytozome.jgi.doe.gov/pz/portal.html) and National Biotechnology Information Center database (National Center of Biotechnology Information, www.ncbi.nlm.nih.gov/) were used for getting genetic information. BioXM 2.6 software was used to predict the molecular weight and isoelectric point of the gene, and GSDS (http://gsds.cbi.pku.edu.cn/) online program was used to predict the gene structure. Neighbor-joining phylogenetic trees were generated using the MEGA 5.1 program (Tamura et al., 2011). ClustalX 1.83 and GeneDoc were used for multiple alignments.

### Subcellular Localization of *GmWRKY46*

The complete ORF sequences of *GmWRKY46* without a stop codon (1077 bp) were fused to the N-terminal of GFP reporter protein of *pJIT166* vector driven by CaMV35S promoter, generating a fusion construct *pJIT166-GmWRKY46*-*GFP*. The specific primers are listed in Supplementary Table 1. The fusion (*pJIT166-GmWRKY46-GFP*) and control (*pJIT166-GFP*) constructs were transformed into *Arabidopsis* protoplasts, respectively. *Arabidopsis* protoplast preparation and transformation were performed as described previously (Li et al., 2020). The GFP fluorescence in the transiently expressing protoplasts was imaged using a Zeiss LSM780 camera (Carl Zeiss, SAS, Jena, Germany).

### Assays of Transcription-Activating Activity in Yeast

The full-length CDS of *GmWRKY46* was cloned into the pGBKT7 vector to create pGBKT7-*GmWRKY46* fusion vector, and the specific primers are listed in Supplementary Table 1. The fusion and pGADT7 vector were co-transformed into Y2HGold yeast. The transformants were screened on medium lacking Leu and Trp (SD/–Trp–Leu) or on medium lacking Leu, Trp, His, and adenine hemisulfate salt (SD/–Trp–Leu–His–Ade). Meanwhile, the empty vector was transformed as the control.

### Plant Materials and Growth Conditions

The soybean genotype Williams 82 was used in these experiments. Seeds of soybean were germinated on vermiculite medium containing Hoagland's nutrient solution in a growth chamber (16 h light, 30°C/8 h dark, 20°C). Seven-day-old seedlings (removal of cotyledons) were transferred to Hoagland's nutrient solution and treated with two Pi levels (Pi-deficient, 2.5 μM KH_2_PO_4_; Pi-sufficient, 1 mM KH_2_PO_4_) for 11 days. At 10th day, the Pi-deficient group was resupplied with 1 mM Pi for 1 day, called R1d. The nutrient solution was refreshed every 3 days. Leaves and roots were harvested at the indicated times after initiation of Pi starvation treatment. Other soybean tissues were also collected at specific times. All samples were stored at −80°C prior to RNA extraction.

### Reverse Transcription Quantitative Real-Time PCR

Reverse transcription quantitative real-time PCR (RT-qPCR) used three biological replicates, each containing three independent plants. Total RNA was extracted from the plant tissue using the plant RNA Extract Kit (TIANGEN Biotech, Beijing, China). The cDNA was synthesized with the HiScript II Q RT SuperMix (+gDNA wiper) for qPCR (Vazyme Biotech, Nanjing, China). The RT-qPCR was performed using a CFX96 Touch (Bio-Rad, Hercules, USA) with AceQ® qPCR SYBR Green Master Mix (Vazyme Biotech, Nanjing, China). The relative level of expression was calculated using the formula 2^−ΔCt^ or 2^−ΔΔCt^. The primers used for RT-qPCR analyses are listed in Supplementary Table 2.

### Development of Transgenic *Arabidopsis*

The full-length target gene coding sequence (CDS) without the stop codon was inserted after the CaMV35S promoter, resulting in overexpression of the target gene constructs with GFP tags. The fusion constructs were confirmed by sequencing and then transformed into wild-type *Arabidopsis* (Col-0) plants. The gene-specific primers are listed in Supplementary Table 1. *Arabidopsis* plants were grown in a controlled environment at 23°C in a 16 h light/8 h dark photoperiod. The transformation and screening methods of *Arabidopsis* were performed as described previously (Li et al., 2020). Seeds used for phenotypic assays were harvested in the same environment. Homozygous T3 or T4 seeds were used.

### RNA-Seq Library Construction, Sequencing, and Data Analysis

The seeds of WT and *GmWRKY46*-overexpressing transgenic *Arabidopsis* lines were grown on Hoagland's nutrient solution Phytagel plates containing 1 mM Pi, 0.2 mM Pi, and 62.5 μM Pi for 20 days. Whole seedlings of WT and transgenic *Arabidopsis* plants under three Pi conditions were collected, respectively, for total RNA isolation and RNA-seq analysis. Three independent biological replications were included for RNA-seq, and each biological replication contained five seedlings. Library construction was carried out by GENE DENOVO (Guang Zhou, China). The qualified cDNA libraries were ultimately sequenced by an Illumina HiSeq^TM^ 2500 instrument.

Gene expression was calculated using Fragments Per Kilobase of transcript per Million mapped reads (FPKM) (Griffith et al., 2015) and was compared between transgenic plants and WT control under three Pi conditions. Clustering software was used to perform cluster analysis of gene expression patterns. Assessment of RNA-seq quality, screening of differential expression genes (DEGs), expression pattern analysis, Gene Ontology (GO), and Kyoto Encyclopedia of Genes and Genomes (KEGG) analysis of DEGs were carried out by GENE DENOVO (Guang Zhou, China). To identify differentially expressed genes across samples or groups, the edgeR package (http://www.r-project.org/) was used, and genes with a fold change ≥2 and a false discovery rate (FDR) <0.05 in comparison were listed as significant DEGs (Trapnell et al., 2012). For GO analysis, all DEGs were mapped to GO terms in the GO database (http://www.geneontology.org/), gene numbers were calculated for every term, and significantly enriched GO terms in DEGs compared with the genome background were defined by hypergeometric test. For KEGG analysis, pathway enrichment analysis identified significantly enriched metabolic pathways or signal transduction pathways in DEGs compared with the whole-genome background, and the calculating formula was the same as that in GO analysis. The raw transcriptome reads were submitted to the NCBI Sequence Read Archive (SRA) database under accession: PRJNA724748.

### Yeast One-Hybrid Assay and Chromatin Immunoprecipitation

Promoter fragments were obtained and analyzed through NCBI (https://www.ncbi.nlm.nih.gov/), and specific primers were designed for each W-box motif for yeast one-hybrid (Y1H) assay (Supplementary Table 1) and chromatin immunoprecipitation (ChIP-qPCR) (Supplementary Table 3). For Y1H, first, the *GmWRKY46* CDS without the stop codon was integrated into the sites of the pGADT7 to generate the effector vector pGADT7-*GmWRKY46*. The specific primers are listed in Supplementary Table 1. Then, the F1–F4 fragment from the AED1 (APOPLASTIC, EDS1-DEPENDENT 1) promoter region was integrated into the sites of the pAbAi to generate a reporter vector pAbAi-F1 to F4, respectively. Y1H assay was used to examine the interaction of *GmWRKY46* and the AED1 promoter fragment according to the manual provided by Matchmaker Gold Y1H Library Screening System (Clontech, Dalian, China). The yeast cells co-transformed with the prey, and either of the baits was cultured for 60 h on SD/-Ura/-Leu medium added with or without 300 ng/ml Aureobasidin A (AbA). For ChIP-qPCR, because the vector that we used to create the transgenic *Arabidopsis* has a GFP tag, the *GmWRKY46*-overexpressing transgenic *Arabidopsis* can be directly used for ChIP. ChIP assay was performed using the EpiQuikTM Plant ChIP Kit (Epigentek) in accordance with the instructions of the manufacturer. GFP Tag Monoclonal Antibody (Proteintech, Cat. No. 66002-1-Ig) was used to label the antibody. qPCR was performed with immunoprecipitated genomic DNA fragments, and enrichment was calculated as the ratio of immunoprecipitation to input and WT as control.

### Induction of Transgenic Soybean Hairy Roots

The overexpression vector and empty vector were separately introduced into *Agrobacterium rhizogenes* K599, and the bacterium was used to infect 5 days old soybean seedling (Williams 82) hypocotyls by injection (Attila et al., 2007). About 14 days later, hairy roots were generated at the infected site. When transgenic hairy roots grew to approximately 8–10 cm long, a small part was harvested for RT-qPCR identification. The original main roots of the identified plants were removed and recovered in Hoagland's nutrient solution for 2 days, then the primary roots were cut off and transferred to Hoagland's vermiculite medium containing 1 mM Pi (Pi-sufficient) or 2.5 μM Pi (Pi-deficient) for 14 days. Each transgenic composite plant represented one independent transgenic line, and six independent transgenic lines were included for each Pi treatment. One independent soybean composite plant with transgenic hairy roots was considered a semi-biological replicate. A total of three replicates were included in this experiment.

### Measurement of Total P and Soluble Pi Concentration

For the plant total P concentration assay, fresh soybean plant samples were heated at 75°C until completely dry and then ground into powder separately. Approximately 0.1 g of dry plant sample was weighed and digested by 2 ml HNO_3_. After cooling, the digested samples were diluted to 25 ml with distilled water. Then, the concentration of P in the solution was determined by Optima 8000 ICP-OES (PerkinElmer, USA). For the measurement of soluble Pi concentration, the Tissue Inorganic Phosphorus Content Detection Kit (Solarbio, Beijing, China) was used. All experiments included three biological replicates.

### Analysis of Root Development

The root development of soybean was analyzed using the scanner (Epson, Expression 21000XL, Japan) with a root analysis system (WinRHIZO 2020). Photography and root analysis of transgenic *Arabidopsis* were described previously (Li et al., 2020), seedlings were grown on square petri dishes under Pi-sufficient (1 mM Pi) or Pi-deficient (0.2 mM Pi and 62.5 μM Pi) condition for 15 days. All experiments included three biological replicates.

### Statistical Analysis

The two-tailed Student's *t*-test (*P* ≤ 0.05) was used to identify the statistical significance of any differences observed. The Microsoft Excel 2019 for Windows V10 was used for all statistical analyses.

## Results

### Isolation and Bioinformatic Analysis of *GmWRKY46*

**Figure 1:**
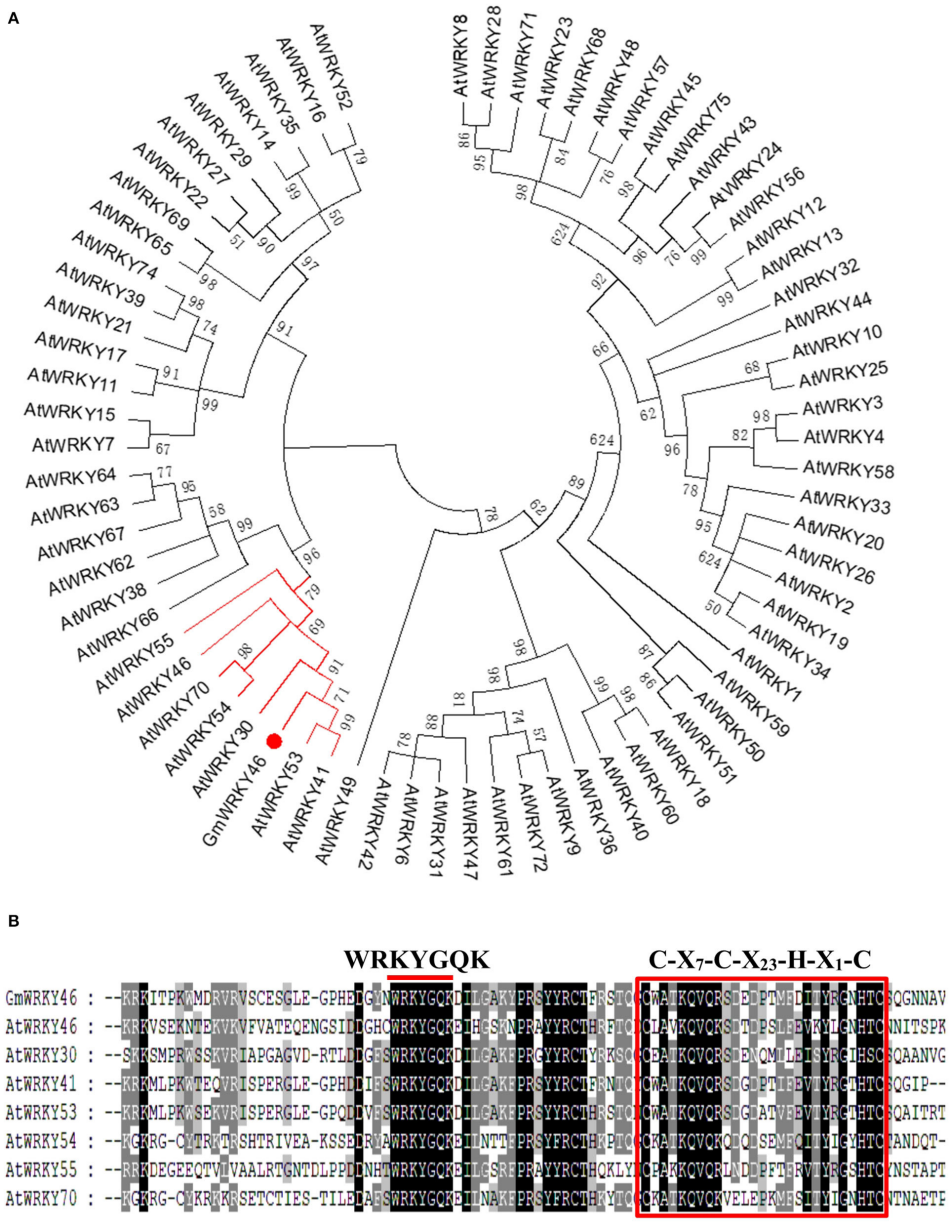
Homology characterization and structure of GmWRKY46. **(A)** a phylogenetic comparison was made between the sequences of the GmWRKY46 protein and the *Arabidopsis* WRKY family proteins. The red line showed proteins with high homology to GmWRKY46. **(B)** Multi-sequence alignment between GmWRKY46 and its highly homologous *Arabidopsis* WRKY protein. The red bar above the sequences represented the highly conserved WRKYGQK domain. The red square represented the N-terminal C-X_7_-C-X_23_-H-X_1_-H zinc-finger structure.

Studies have shown that *AtWRKY46* regulates lateral roots development in *Arabidopsis thaliana* under osmotic/salt stress (Ding et al., 2015), and we found in a report that *GmWRKY46* was highly expressed in soybean roots (Song et al., 2016). In osmotic/salt stress conditions, lateral root development is significantly reduced in loss-of-function *wrky46* mutants, while overexpression of *WRKY46* enhances lateral root development (Ding et al., 2015). In view of the close correlation between lateral roots development and Pi starvation response of plants (Péret et al., 2011), it can be speculated that *GmWRKY46* may be involved in the response of soybean to Pi starvation. *GmWRKY46* (GLYMA_08G021900) is located on chromosome 8 of soybean, position 1763171-1764910. Bioinformatics analysis showed that *GmWRKY46* contained a 1080 bp open reading frame (ORF) and encoded a predicted polypeptide of 359 amino acids with a calculated molecular mass of 89.7 kDa and an isoelectric point of 4.8. A phylogenetic tree was constructed based on the GmWRKY46, and using a total of 72 WRKYs from *Arabidopsis* showed that GmWRKY46, AtWRKY30, AtWRKY41, AtWRKY46, AtWRKY53, AtWRKY54, AtWRKY55, and AtWRKY70 had high homology (Figure 1A). Multiple sequence alignment analysis revealed that the N-terminal of GmWRKY46 protein contained a WRKYGQK domain and a C-X_7_-C-X_23_-H-X_1_-H zinc-finger structure (Figure 1B), so GmWRKY46 belonged to the group III subfamily of the WRKY TF family (Chen et al., 2017a).

### Analysis of Subcellular Localization and Transcription-Activating Activities of GmWRKY46

**Figure 2:**
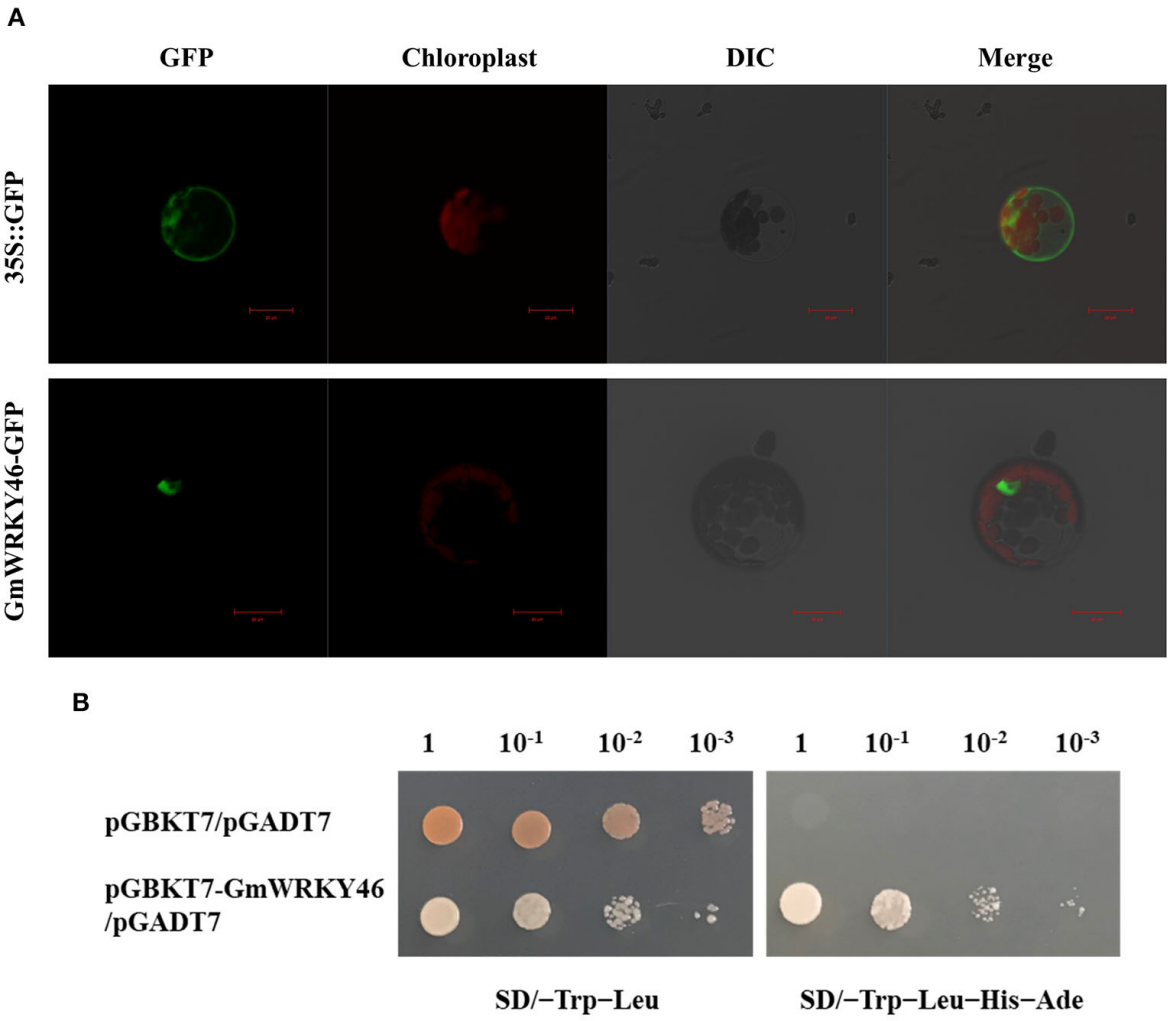
Analysis of subcellular localization and transcription-activating activities of GmWRKY46. **(A)** Subcellular localization of GmWRKY46-GFP and 35S::GFP protein in *Arabidopsis* protoplasts. Scale bar = 20 μm. **(B)** Y2H assay for transcription-activating activities of GmWRKY46. Yeast cells co-transformed with pGBKT7-GmWRKY46/pGADT7 were grown on selective media SD/-Trip-Leu and SD/-Trip-Leu-His-Ade. Empty pGBKT7/pGADT7 was used as control.

To investigate the subcellular localization of GmWRKY46, the recombinant constructs of the GmWRKY46-GFP fusion gene and GFP alone were introduced into *Arabidopsis* mesophyll protoplasts, respectively. Under a confocal laser microscope, the fluorescence of GMWRKY46-GFP was specifically detected in the nucleus, whereas the control construct was present throughout the whole cell (Figure 2A). These results indicate that GmWRKY46 was localized in the nucleus and was consistent with the predicted function as a transcription factor (TF).

Transcription factor is a protein molecule, and it usually contains a DNA-binding domain and a transcription activation domain. In order to verify whether GmWRKY46 has transcriptional activator activity, we used the yeast two-hybrid (Y2H) system. As shown in Figure 2B, the yeast cells transformed with the fusion construct pGBKT7-GmWRKY46 could grow normally in both SD/–Trp–Leu medium and SD/–Trp–Leu–His–Ade medium, while the growth of the control was inhibited in the SD/–Trp–Leu–His–Ade medium. These results indicated that the fusion proteins of GmWRKY46 could activate both HIS3 and AED2 reporter genes and could therefore act as transcriptional activators in yeast cells.

### Expression Patterns of *GmWRKY46*

**Figure 3:**
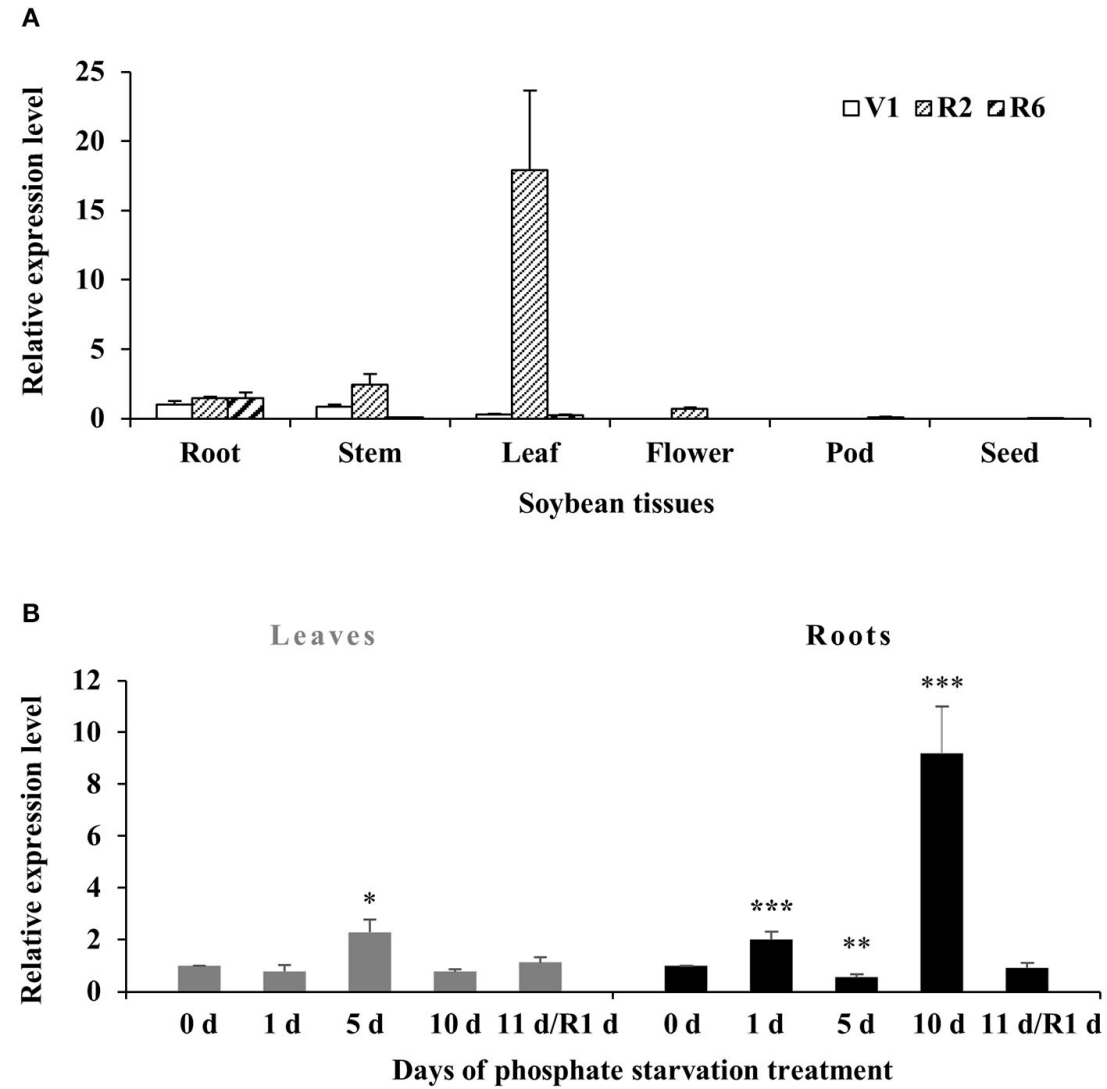
Expression patterns of *GmWRKY46* in soybean. **(A)** The expression of GmWRKY46 in roots, stems, leaves, flowers, pods, and seeds at the first trifoliate stage (V1), full bloom stage (R2), and full seed stage (R6). **(B)** Time course of the expression level of *GmWRKY46* in leaves and roots. 0, 1, 5, 10, and 11 days, duration of Pi starvation (days); Recovery 11 days (R1d), 10 days of Pi starvation followed by 1 day on Pi-sufficient substrate. The expression level at 0 h was set as 1, and data represented means ± SD of three replicates. Data significantly different from the corresponding controls were indicated (Student's *t*-test, *P < 0.05; **P < 0.01; ***P < 0.001).

To investigate the transcript levels of *GmWRKY46* in specific tissues, the total RNA was extracted from root, stem, leaf, flower, pod, and the seed of soybean plants at first trifoliate (V1), full bloom (R2), and full seed (R6) stages. RT-qPCR analysis showed that the expression of *GmWRKY46* remained stable in the roots of the three stages. It should be noted that *GmWRKY46* expression in the leaves of the R2 stage was greatly increased compared with the other two stages. In addition, we noted that almost no expression of *GmWRKY46* was detected in all tissues at the R6 stage, except the roots (Figure 3A).

Then, the expression patterns of *GmWRKY46* were analyzed by RT-qPCR under Pi starvation in roots and leaves. As shown in Figure 3B, there was no significant change in *GmWRKY46* transcription level in leaves after Pi starvation except for the increase on the 5th day. In roots, *GmWRKY46* transcript levels quickly accumulated on the 1st day after Pi starvation, exhibited a dramatic decrease on the 5th day and then induced more than 9-fold of the normal level on the 10th day (Figure 3B). After resupplying Pi, the transcript level of *GmWRKY46* was close to the normal Pi level in both leaves and roots. These results indicated that *GmWRKY46* was involved in soybean response to Pi starvation.

### *GmWRKY46* Enhancement of the Tolerance to Pi Starvation May Depend Partly on Improving Root Development in Transgenic *Arabidopsis*

To determine the functions of *GmWRKY46* in plant tolerance to Pi starvation, we attempted to use transgenic *Arabidopsis* due to the difficulty in obtaining transgenic soybean. Four *GmWRKY46*-overexpressing transgenic lines were identified by PCR and RT-qPCR (Supplementary Figures 1A,B), and three of them with higher expression levels of *GmWRKY46* were selected for further studies (Supplementary Figure 1B). Two-week-old seedlings of transgenic *Arabidopsis* lines and the wild type (WT) were grown in the greenhouse for 20 days under 1 mM Pi (Pi-sufficient) and 62.5 μM Pi (Pi-deficient) conditions. Under Pi-sufficient condition, the growth period of all plants was found to be the same, with all plants bolting (Supplementary Figure 2). However, the growth of WT plants was inhibited under Pi-deficient conditions, while the bolting of *GmWRKY46*-overexpressing transgenic *Arabidopsis* was not affected (Supplementary Figure 2). These results suggested that overexpression of *GmWRKY46* enhanced tolerance to Pi starvation in transgenic *Arabidopsis*.

**Figure 4:**
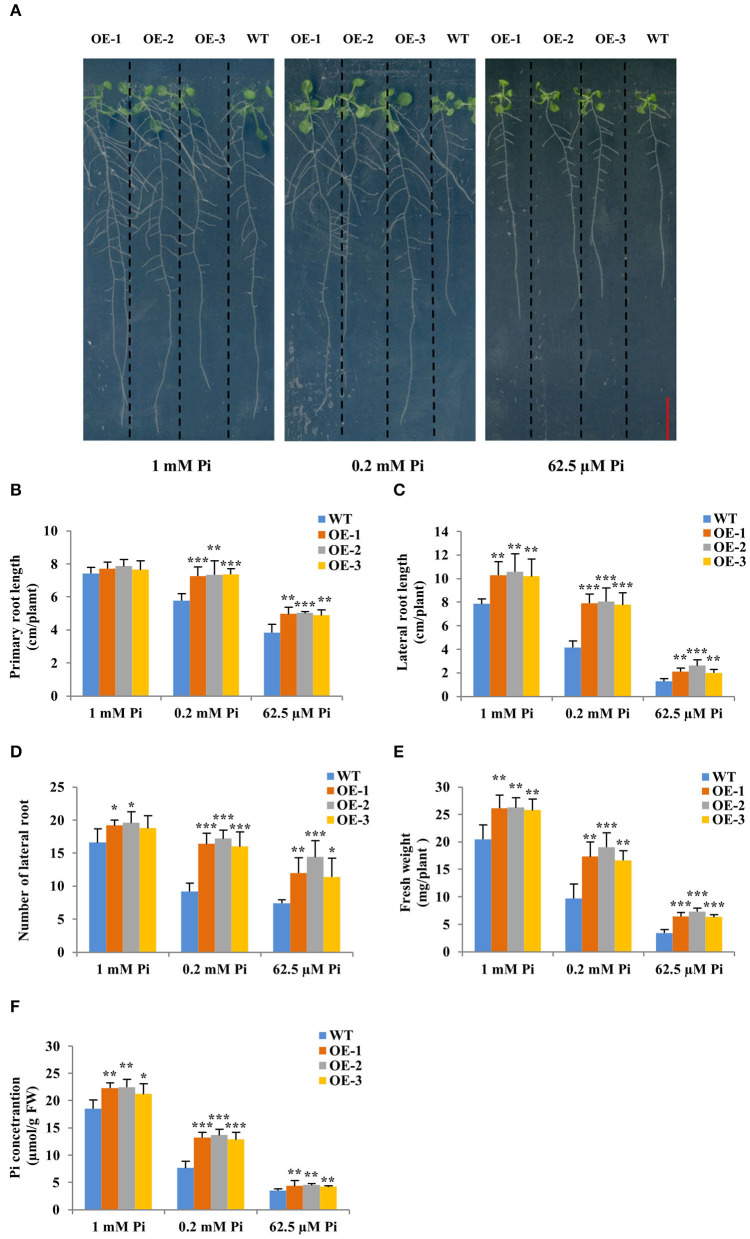
Overexpression of *GmWRKY46* enhanced Pi starvation tolerance and lateral roots growth in transgenic *Arabidopsis*. **(A)** WT and GmWRKY46-overexpressing transgenic *Arabidopsis* were grown under three different Pi concentrations (Pi-sufficient, 1 mM Pi; Pi-deficient, 0.2 mM Pi and 62.5 μM Pi) for 15 days on vertically oriented petri plates, respectively. Primary root length **(B)**, lateral root length **(C)**, number of lateral root **(D)**, fresh weight **(E)**, and Pi concentration **(F)** were determined in WT and transgenic *Arabidopsis* plants on the 15th day. Values were mean ± SD (*n* = 3), and asterisks showed that the values were significantly different between the transgenic lines and the WT (Student's *t*-test, *P < 0.05, **P < 0.01, ***P < 0.001).

Then, the three *GmWRKY46*-overexpressing transgenic *Arabidopsis* lines and WT were planted on vertical Phytagel plates containing three different Pi concentrations medium (Pi-sufficient, 1 mM Pi; Pi-deficient, 0.2 mM Pi, and 62.5 μM Pi, respectively) and cultured for 20 days to observe their phenotypes. We found that short-term Pi deficiency significantly inhibited the growth of *Arabidopsis*, especially the root growth, and the lower the environmental Pi concentration, the more obvious this inhibition (Figure 4A). However, this inhibitory effect was weakened in transgenic *Arabidopsis* (Figure 4A). We found that the primary and lateral root length of transgenic lines was significantly longer, and the lateral root number of transgenic lines was significantly more than those of WT under Pi-deficient conditions (Figures 4B–D). Furthermore, the lateral root length of transgenic lines was also significantly longer than that of WT under Pi-sufficient condition (Figure 4B). The lateral root number of all transgenic lines was significantly more than that of WT under Pi-sufficient condition, except OE-3 (Figure 4D), which we thought might be probably due to the relatively low expression of *GmWRKY46* in OE-3. There was no significant difference between the primary length of the transgenic lines and the WT under Pi-sufficient condition (Figure 4B). Additionally, the fresh weight and Pi concentration of the transgenic lines were significantly higher than those of the WT under three Pi level conditions (Figures 4E,F). Together, these results suggested that overexpression of *GmWRKY46* significantly improves the uptake of Pi in transgenic *Arabidopsis* by promoting root system development, and at the same time, its positive effect on lateral root development might be independent of the Pi level of the environment.

### Differentially Regulated Genes Identified From RNA-seq Analysis

The Pi starvation tolerance phenotype of transgenic *Arabidopsis* also may be due to gene expression changes caused by the *GmWRKY46*. WRKY transcription factors were generally thought to regulate the expression of their target genes by binding to the W-box(es) in their target gene promoters (Rushton et al., 2010), and hence, it was reasonable to speculate that *GmWRKY46* might recognize heterologous promoters from a similar protein structure/DNA sequence interaction. To examine changes in gene expression patterns, RNA-seq analysis was conducted on 20-day-old plants under 1 mM Pi, 0.2 mM Pi, and 6.25 μM Pi conditions and compared the *GmWRKY46*-overexpressing *Arabidopsis* against the WT control. Under 1 mM Pi condition, the expression profile of *GmWRKY46*-overexpressing *Arabidopsis* compared with the WT control was hereafter referred to as comparison 1 (C1); likewise, under 0.2 mM Pi condition, OE-*GmWRKY46* compared with the WT was C2 and under 6.25 μM Pi condition was C3.

**Figure 5:**
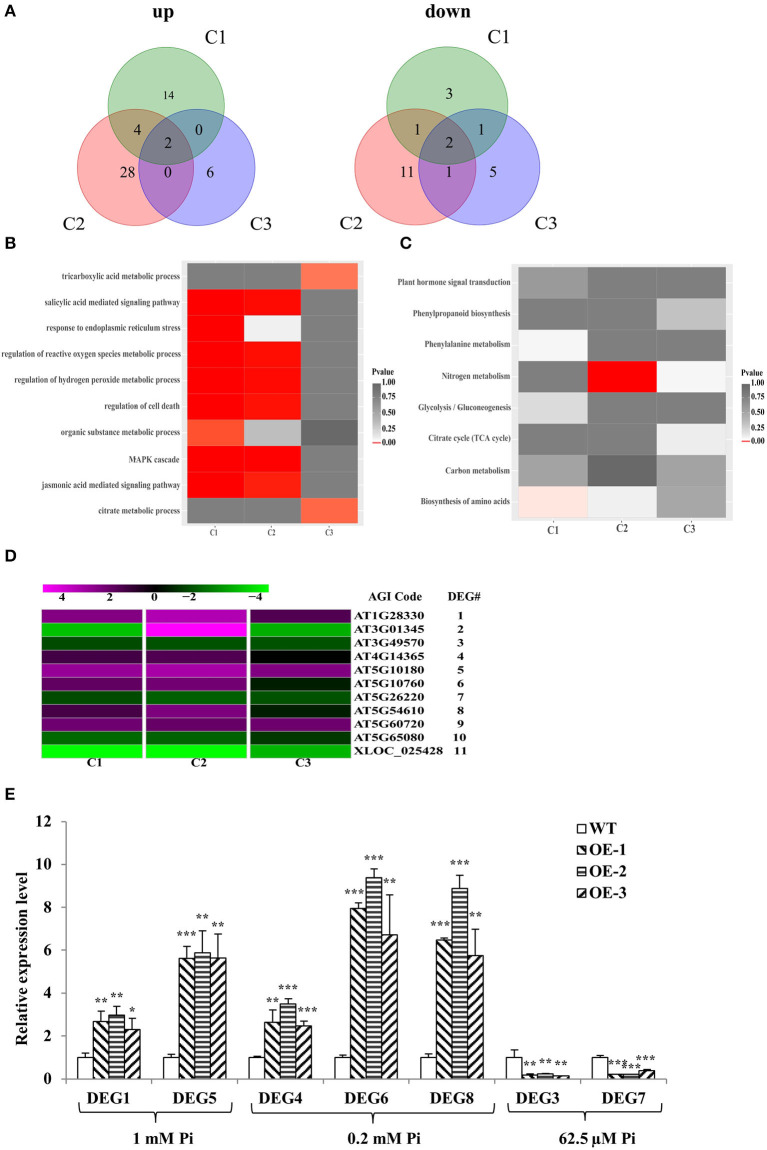
RNA-seq analysis and RT-qPCR verification. **(A)** Venn diagrams of all genes exhibiting upregulated or downregulated expression between WT and *GmWRKY46*-overexpressing *Arabidopsis* plants. C1, C2, and C3 represent different levels of Pi. **(B,C)** Results of the GO enrichment analysis **(B)** and KEGG pathway enrichment analysis **(C)** of the DEGs between WT and *GmWRKY46*-overexpressing *Arabidopsis* plants. **(D)** Heat map of clustering analysis of the 11 DEGs in the intersection of C1, C2, or C3. Expression ratios are shown as log_2_ values. Magenta represented increased expression; green represented decreased expression; black represented no difference in expression compared with control. **(E)** Expression patterns of 7 DEGs candidates. Vertical axis showed fold enrichment of relative transcript levels between transgenic and WT plants. Expression was normalized to that of Actin2/8. Data were means ± SD (*n* = 3). Data significantly different from the WT were indicated (Student's *t*-test, *P < 0.05, **P < 0.01, ***P < 0.001).

There were 27 differential expression genes (DEGs) in C1, 49 DEGs in C2, and 17 DEGs in C3 (Supplementary Table 4); 20, 34, and 8 genes were upregulated in C1, C2, and C3, respectively; and 7, 15, and 9 genes were downregulated in C1, C2, and C3, respectively (Figure 5A). DEGs were then subjected to enrichment analysis of Gene Ontology (GO) and Kyoto Encyclopedia of Genes and Genomes (KEGG) (Supplementary Table 4). The results showed that the DEGs were commonly involved in energy metabolism (tricarboxylic acid and citrate metabolic process, organic substance metabolic process, and nitrogen and carbon metabolism), stress responses (response to endoplasmic reticulum stress, regulation of cell death, regulation of hydrogen peroxide and reactive oxygen species metabolic process, biosynthesis of amino acids, phenylalanine metabolism, and MAPK cascade), and plant hormone signal transduction (salicylic acid and jasmonic acid-mediated signaling pathway) (Figures 5B,C). Given that 11 DEGs were common to C1 and C2 or C1 and C3 or C2 and C3, we narrowed the focus to this smaller group. The 11 members were named DEG1 to DEG11 from top to bottom in the clustering analysis map (Figure 5D). Among these 11 DEGs, five are annotated to have molecular functions or transporter activity (Supplementary Table 5). In addition, to verify the RNA-seq analysis results, seven DEGs were chosen and evaluated in a qRT-PCR assay with the same tissues used for the RNA-seq analysis (Figure 5E). The expression patterns of the selected genes were consistent with the RNA-seq data.

### Overexpression of *AtAED1* (DEG6) Enhanced Tolerance to Pi Starvation in Transgenic *Arabidopsis*

**Figure 6:**
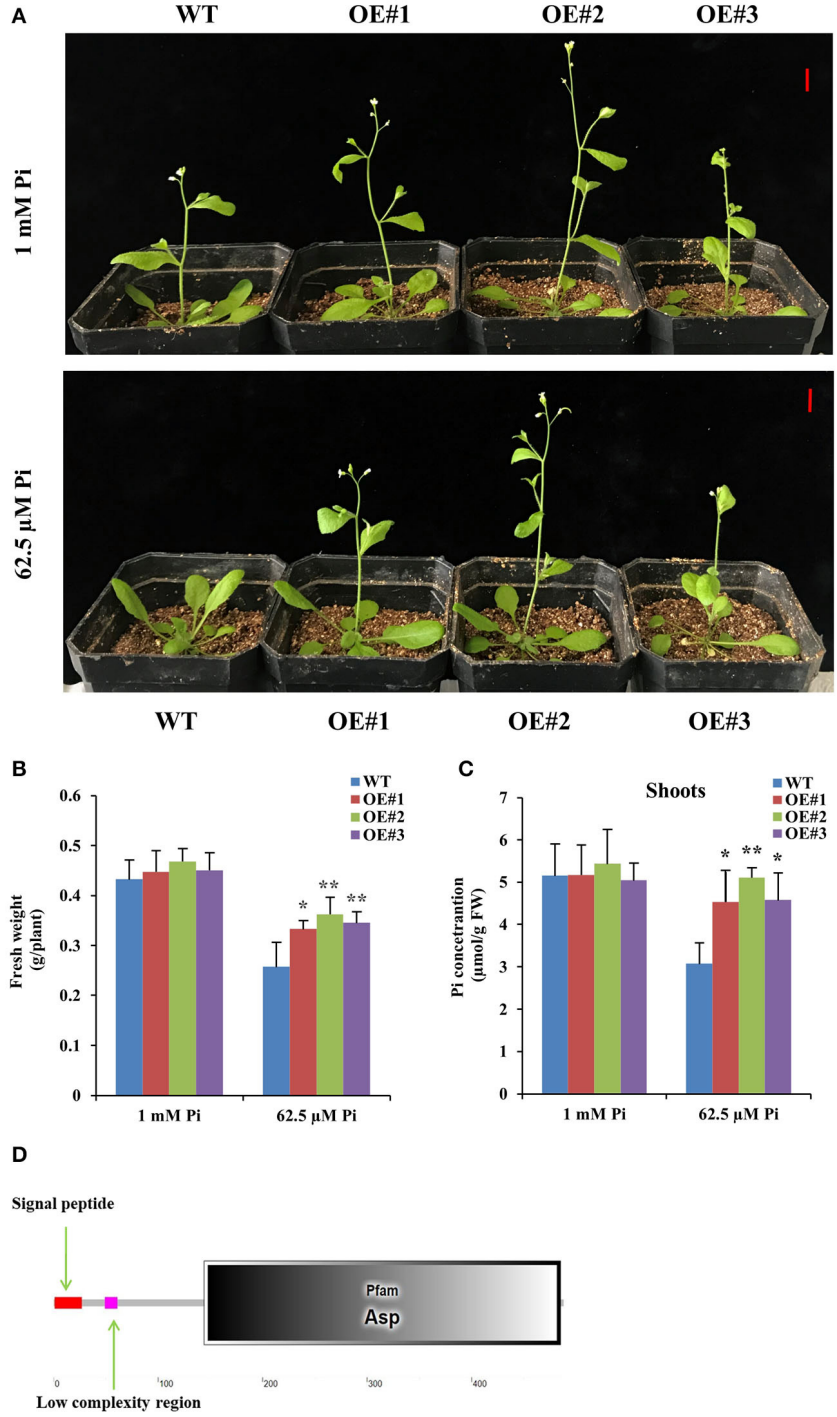
Overexpression of *DEG6* enhanced tolerance to Pi starvation in transgenic *Arabidopsis*. **(A)** Two-week-old seedlings were grown in the greenhouse for 20 days under 1 mM Pi (Pi-sufficient) and 62.5 μM Pi (Pi-deficient) conditions. WT, wild type *Arabidopsis* lines; OE#1 to 3, independent *DEG6*-overexpresing *Arabidopsis* lines. Bars: 1 cm. **(B, C)** The fresh weight and shoots Pi concentration of WT and *DEG6*-overexpresing *Arabidopsis* on the 20th day. Data means ± SD (*n* = 3). Asterisks indicated significant differences between transgenic *Arabidopsis* and WT (Student's *t*-test, *P < 0.05; **P < 0.01). **(D)** Protein domain analysis of DEG6.

We obtained 11 DEGs through cluster analysis, among which 6 DEGs had upregulated performance in at least two cases of C1, C2, and C3 (Figure 4D), so we further focused on the analysis of these 6 DEGs (DEG1, DEG4, DEG5, DEG6, DEG8, and DEG9). Interestingly, promoter analysis showed that the promoter of the 6 DEGs contained at least one W-box motif, respectively (Supplementary Figure 3), suggesting that all of them might be regulated by *GmWRKY46*. So, we constructed transgenic *Arabidopsis* with overexpression of the 6 DEGs, respectively. At least two independent transgenic *Arabidopsis* lines were obtained for each gene and verified by PCR or RT-qPCR. Here, we only showed the data of *DEG6* (OE#1, OE#2, and OE#3) (Supplementary Figure 4). Two-week-old seedlings of all transgenic *Arabidopsis* lines and the WT were grown in the greenhouse for 20 days under Pi-sufficient 1 (mM Pi) and Pi-deficient (62.5 μM Pi) conditions. We found that bolting of transgenic *Arabidopsis* with overexpression of the *DEG6* was not inhibited by Pi starvation when compared with WT and was similar to the phenotype observed for *GmWRKY46* overexpression (Figure 6A, Supplementary Figure 2). Further data analysis showed that *DEG6*-overexpressing transgenic *Arabidopsis* had significantly higher fresh weight and shoot Pi concentrations than WT under low Pi condition (Figures 6B,C). However, *DEG6*-overexpressing transgenic *Arabidopsis* did not produce a root phenotype like that of *GmWRKY46*-overexpressing transgenic *Arabidopsis* in plate growth experiments (data not shown). Bioinformatics analysis showed that DEG6 was a eukaryotic aspartyl protease family protein with an Asp domain (Figure 6D), known as AED1 (Breitenbach et al., 2014). These results suggested that overexpression of *AtAED1* enhanced tolerance to Pi starvation in transgenic *Arabidopsis*.

### GmWRKY46 Directly Interacts With the Promoter of *AtAED1 (DEG6)*

**Figure 7:**
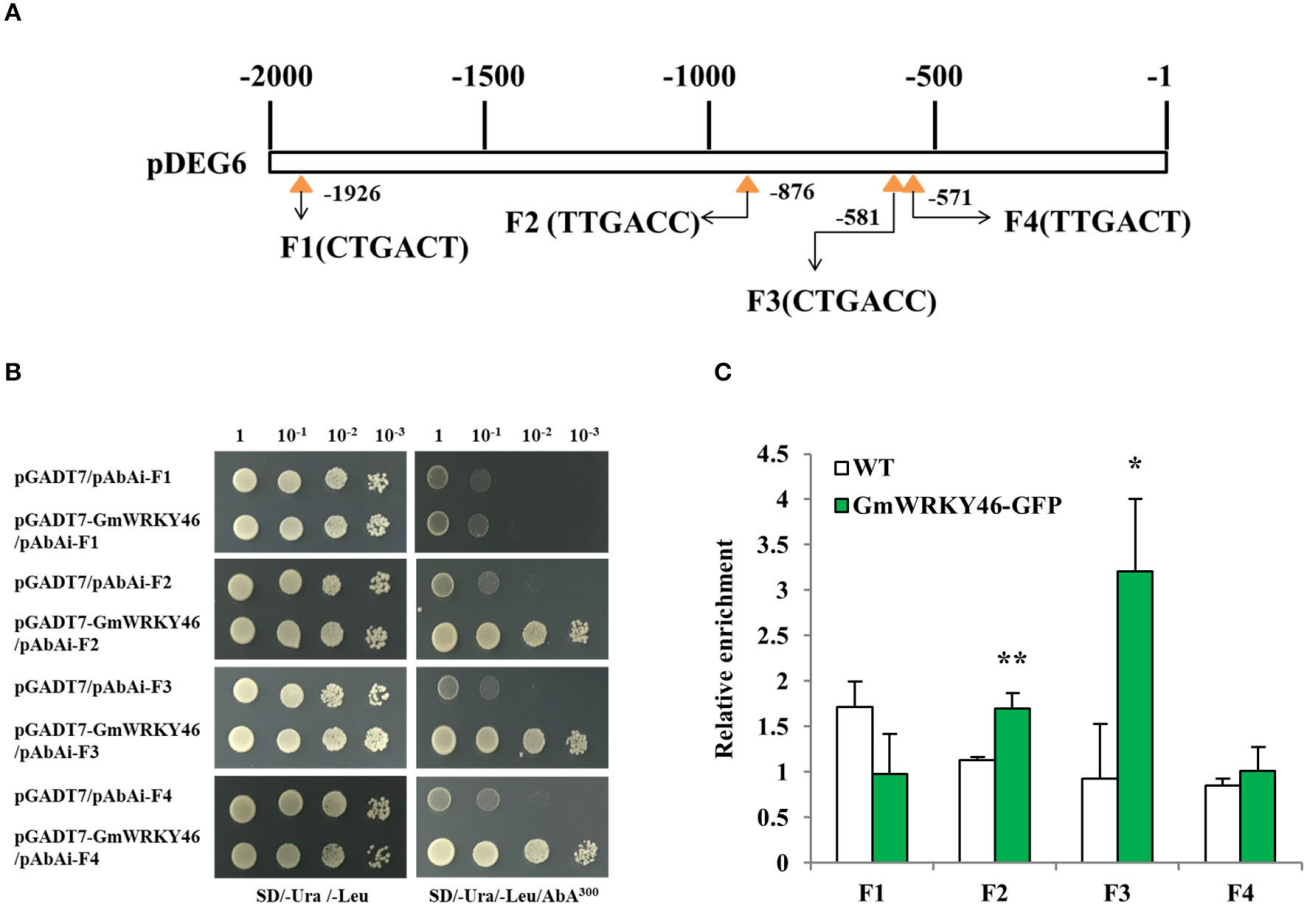
GmWRKY46 binds to *DEG6* promoter region *in vivo* and *in vitro*. **(A)** Diagram of the 2000-bp promoter region of DEG6 showing the relative positions of the W-boxes. Four W-boxes F1, F2, F3, and F4 were marked by yellow triangle. Numbers indicated the position of starting nucleotide of each W-box relative to translation start. **(B)** GmWRKY46 binds to the *DEG6* promoter region in the Y1H assay. Yeast cells were transformed with a bait vector containing a promoter fragment F1, F2, F3, or F4 fused to pAbAi vector, and a prey vector containing GmWRKY46 fused to pGADT7 vector. Yeast cells were grown in liquid medium to an OD600 of 1.0 and diluted in a 10× dilution series (10^−1^-10^−3^). From each dilution, 5 μl was spotted onto SD/-Ura/-Leu medium to select for plasmids, and SD/-Ura/-Leu supplemented with 300 ng/ml Aureobasidin A (AbA) to select for interaction. Empty pGADT7 was used as control. **(C)** ChIP-qPCR analysis of GmWRKY46 binding to the DEG6 promoter region. *Arabidopsis* seeds of WT and Pro35S:GmWRKY46-GFP transgenic plants were germinated in medium and supplied with sufficient Pi. The whole plants were harvested for ChIP analysis. Enriched DNA fragments (F1 to F4) in the DEG6 promoter were quantified using RT-qPCR. Enrichment was calculated as the ratio of immunoprecipitation to input. Values represent means ± SD (*n* = 3). Data significantly different from the control are indicated (Student's *t*-test, *P < 0.05; **P < 0.01).

To determine whether *DEG6* was the target gene of *GmWRKY46*, four W-boxes (F1–F4) in the DEG6 upstream 2000-bp long promoter fragment were used for Y1H assay to examine the interaction between GmWRKY46 and the *DEG6* promoter (Figure 7A). Therefore, a 48 bp fragment containing three copies of F1–F4 with 5 bp promoter sequence on both sides, respectively, was used as bait and cloned into the reporter vector, while GmWRKY46 was used as prey. The yeast cells of control (pGADT7/pAbAi-F1 ~ F4) and those co-transformed with prey (pGADT7-GmWRKY46)-bait (pAbAi-F1 ~ F4) grew normally in the screening medium (Figure 7B). However, when 300 ng/ml AbA was added, growth of the control and pGADT7-GmWRKY46/pAbAi-F1 was completely inhibited, while pGADT7-GmWRKY46/pAbAi-F2, pGADT7-GmWRKY46/pAbAi-F3, and pGADT7-GmWRKY46/pAbAi-F4 transformants survived (Figure 7B). These results suggested that GmWRKY46 can specifically bind to three W-box cis-acting elements, F2, F3, and F4 on the *DEG6* promoter *in vitro*, but cannot bind F1.

To further determine whether GmWRKY46 binds the *DEG6* promoter *in vivo*, specific primers were used against the *DEG6* promoter corresponding to fragments F1 ~ F4 for ChIP-qPCR, respectively. The ChIP-qPCR showed that the relative DNA enrichment amount of GmWRKY46-GFP transgenic *Arabidopsis* plants at F2 and F3 was significantly higher than that of WT plants (Figure 7C), and the binding ability between GmWRKY46 and F3 was stronger than that between GmWRKY46 and F2. The results indicated that GmWRKY46 could specifically bind the *DEG6* **(***AtAED1*) promoter in *Arabidopsis*. In addition, we observed that GmWRKY46 and F4 bind in the yeast system, but did not bind *in vivo*. The possible reason might be due to the closeness of F3 and F4 (interval 4 bp), thus influencing each other (Rushton et al., 2010). This interaction may also explain the stronger binding ability of GmWRKY46 and F3 than that of GmWRKY46 and F2.

### Functional Analysis of *GmWRKY46* in Soybean Composite Plants

**Figure 8:**
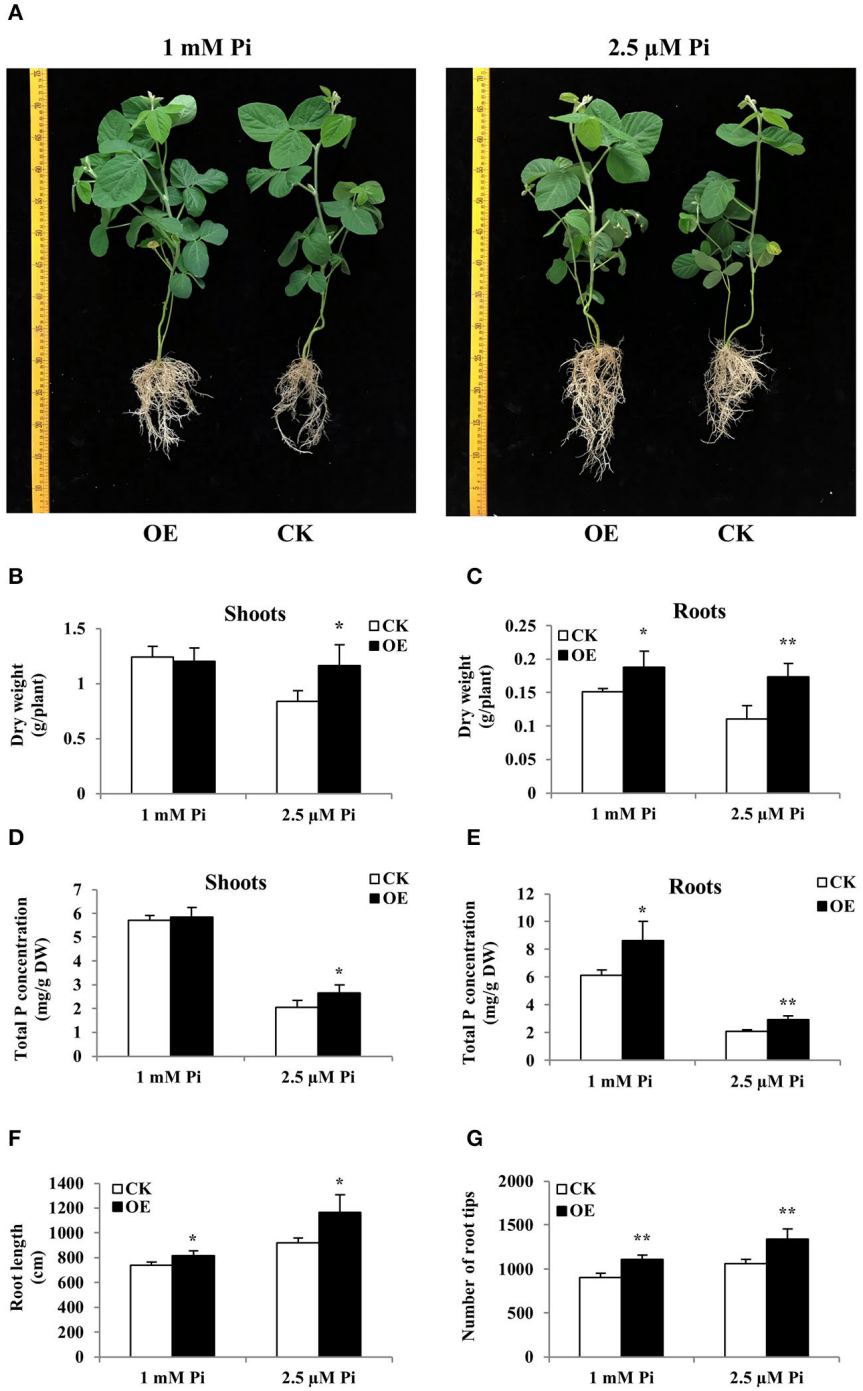
Comparison of control (CK) and *GmWRKY46*-overexpressing composite soybean plants (OE) under two Pi level conditions. Composite soybean plants with transgenic hairy roots were transferred to a nutrient solution containing 1 mM Pi (Pi-sufficient) or 2.5 μM Pi (Pi-deficient). After a further 14 days, shoots and roots were separately harvested for analysis. **(A)** Phenotype of CK and OE. Scale unit: cm. **(B)** Dry weight of shoots. **(C)** Dry weight of roots. **(D)** Total P concentration of shoots. **(E)** Total P concentration of roots. **(F)** Root length of composite plants. **(G)** Number of root tips of composite plants. CK represents the composite plants transformed with the empty vector; OE indicates composite plants with *GmWRKY46*-overexpressing transgenic hairy roots. DW means dry weight. Data are means ± SD of three biological replicates. Asterisks indicate significant differences between OE and CK (Student's *t*-test, *P < 0.05; **P < 0.01).

The “composite plant system” containing transgenic hairy root and their attached WT shoot can be obtained by genetic transformation of hairy roots, providing a rapid and effective method for functional analysis of genes expressed in soybean roots (Guo et al., 2011). Due to the strong response of *GmWRKY46* to the low Pi in the soybean root, the “composite plant system” was used for further studying the functions of *GmWRKY46* in the low Pi tolerance and root development in soybean. Increased expression of *GmWRKY46* in transgenic hairy roots was verified through RT-qPCR analysis (Supplementary Figure 5). The composite soybean plants with transgenic hairy roots were transferred to a nutrient solution containing 1 mM Pi (Pi-sufficient) or 2.5 μM Pi (Pi-deficient). After 14 days of Pi starvation, the control plants showed symptoms of P deficiency, such as small, narrow, and dark green leaves, while the transgenic composite plants with *GmWRKY46*-overexpressing hairy roots were relatively normal (Figure 8A). At the same time, overexpression of *GmWRKY46* in hairy roots of soybean significantly increased the dry weight and total P concentration of transgenic composite plants compared with the control under Pi-deficient condition. More precisely, the dry weight rose by 38% in shoots and by 58% in roots, and the total P concentration rose by 29% in shoots and by 41% in roots under Pi-deficient condition (Figures 8B–E). In addition, overexpression of *GmWRKY46* significantly elongated hairy roots length and increased the number of root tips compared with control plants regardless of Pi supply (Figures 8F,G). Under Pi-sufficient condition, we found that overexpression of *GmWRKY46* resulted in a significant increase in dry weight and total P concentration of roots compared with control plants (Figures 8B–E). The above results implied that the overexpression of *GmWRKY46* in soybean hairy roots could dramatically regulate root development, thus affecting the tolerance of soybean to Pi starvation.

## Discussion

As one of the largest TF families in plants, the WRKY TFs family plays an important role in the regulation of plant growth, development, and defense response mechanisms (Singh et al., 2002). Although *AtWRKY75* was reported as a member of the WRKY TFs family related to Pi acquisition and root development in *Arabidopsis* as early as 2007 (Devaiah et al., 2007), the role of WRKY TFs under Pi starvation in soybean and other higher crops remains unclear. Here, we functionally and mechanically characterize a WRKY TF, GmWRKY46, from soybean under low Pi stress. Overexpression of *GmWRKY46* promoted Pi starvation tolerance and root development in transgenic plants. In addition, we also further demonstrated that GmWRKY46 directly binds to the promoter of *AtAED1*, thereby enhancing the tolerance to Pi starvation in transgenic *Arabidopsis*. Our study provided a basis for the molecular genetic breeding of Pi starvation tolerance in soybean and novel insight into the regulatory pattern mediated by WRKYs in the stress response.

GmWRKY46 belonged to group III subfamily of the WRKY TF family, which was localized in the nucleus and had transcriptional activator activity. Interestingly, most of the *Arabidopsis* WRKY family of genes with high homology to *GmWRKY46* have been found to be associated with abiotic stress. For example, *AtWRKY30* enhanced abiotic stress tolerance during early growth stages in *Arabidopsis* (Scarpeci et al., 2013), *AtWRKY53* negatively regulated drought tolerance by mediating stomatal movement (Sun and Yu, 2015), and *AtWRKY46/54/70* involved in brassinosteroid-regulated plant growth and drought responses (Chen et al., 2017b). Our results showed that Pi starvation strongly induced *GmWRKY46* expression, particularly in roots, suggesting that it may play an important role in the root response to Pi starvation. It should be noted that the expression of *GmWRKY46* in soybean leaves of R2 stage was significantly increased compared with the other two stages, which might indicate that expression of the gene in soybean leaves was developmentally regulated. This expression pattern was like that of *GmWRKY58* and *GmWRKY76*, which were initially expressed at very low levels in leaves, then highly expressed in relatively young leaves, but then rapidly declined in old leaves (Yang et al., 2016b).

*Arabidopsis* has been commonly used in transgenic studies for stress-tolerant genes from crops that are not easy for gene transformation analysis, including soybean (Liao et al., 2008; Niu et al., 2012; Kong et al., 2018). Overexpression of *GmWRKY46* enhanced tolerance to Pi starvation in transgenic *Arabidopsis* plants as revealed from plant growth situation and changes in root development, fresh weight, and Pi concentration after low Pi treatments. Moreover, we further demonstrate the role of *GmWRKY46* in adaptation to Pi starvation in soybean by using a “composite plant system,” which consisted of transgenic hairy roots attached to wild-type shoots for “whole-plant” functional analysis (Guo et al., 2011; Bian et al., 2020). Under Pi-deficient condition, the dry weight and total P concentration of *GmWRKY46*-overexpressing transgenic soybean composite plants were higher than those of the control plants. Root analysis showed that the root length and number of *GmWRKY46*-overexpressing transgenic *Arabidopsis* and transgenic soybean composite plants increased significantly under Pi-deficient conditions. Previous studies have shown that root systems play an important role in Pi uptake (Péret et al., 2011; Jia et al., 2018). Therefore, we speculate that *GmWRKY46* could affect plant P efficiency by regulating root adaptive changes in response to Pi starvation.

Interestingly, the lateral root development of transgenic *Arabidopsis* with *GmWRKY46* overexpression was still better than that of WT under Pi-sufficient condition. We speculated that *GmWRKY46* may regulate lateral root development independently of environmental Pi levels. Because plants get Pi mainly through lateral roots (Péret et al., 2011; Jia et al., 2018), better lateral root development should be one of the reasons *GmWRKY46*-overexpressing transgenic *Arabidopsis* has higher fresh weight and Pi concentration than the WT under Pi-sufficient and Pi-deficient conditions. The experiments with the “composite plant system” further confirmed our deduction. Regardless of Pi supply, overexpression of *GmWRKY46* in hairy roots increased root dry weight, root total P concentration, root length, and root tip number of transgenic soybean composite plants. In previous research reports, similar phenomena have been found in *Arabidopsis* and rice. Under both Pi-sufficient and Pi-deficient conditions, when *AtWRKY75* expression was suppressed, lateral root length and number were significantly increased (Devaiah et al., 2007). *OsMYB4P*-overexpressing transgenic rice had longer lateral roots than the WT, regardless of environmental Pi levels (Yang et al., 2014). However, the dry weight of the shoots and the total P concentration of transgenic soybean composite plants were not different from those of control plants under Pi-sufficient condition. We speculate that this may be a self-protective mechanism of soybean, because excessive accumulation of Pi in the shoots may be harmful to plants (Zhou et al., 2008; Wang et al., 2009). Although overexpression of *GmWRKY46* in the soybean hairy roots led to enhanced root development, which was beneficial to Pi acquisition, the upward transfer of Pi from transgenic roots may be limited by wild-type shoots in the “composite plant system.”

Several studies have suggested that some of the basic functional modules of stress-responsive regulatory networks might be shared among higher plants (Chen and Zhu, 2004). Because WRKY TFs play a strict regulatory role in the specific recognition and binding of W-box or W-box similar sequences on downstream promoters, they have a promising application prospect in crop improvement (Phukan et al., 2016). Although our goal was to find the target gene of *GmWRKY46* in soybean to analyze the associated signaling pathway, due to the difficulty of obtaining transgenic soybean, we regressed back to using transgenic *Arabidopsis* for a first analysis. As expected, the RNA-seq analysis of the overexpression of *GmWRKY46* revealed that there were a lot of differentially expressed genes (DEGs) in transgenic *Arabidopsis* compared with the WT at three Pi levels, with at least 60 of them upregulated. Many DEGs were involved in energy metabolism, stress response, and hormone synthesis and transport, and these biological processes have been found in relation to Pi starvation in previous studies (Woo et al., 2012; O'Rourke et al., 2013). These results suggest that *GmWRKY46* actively activates the response of transgenic *Arabidopsis* to Pi starvation.

In RNA-seq analysis, we set three Pi levels to capture the key target genes more accurately. However, only two genes were found to be co-upregulated at all three Pi levels, which might indicate that our experimental design is not perfect. The gene changes caused by Pi starvation may be transient and rapid, so many potentially relevant target genes may be lost by RNA-seq analysis when sampled after prolonged low Pi stress (Wu et al., 2003). In order to achieve our goal, we extended the scope to 6 DEGs upregulated at any two Pi levels and then overexpressed them in *Arabidopsis*. We found that overexpression of *DEG6* also improved the resistance of transgenic *Arabidopsis* to Pi starvation. Furthermore, Y1H and ChIP-qPCR assays showed that GmWRKY46 binds to the W-boxes on the DEG6 promoter, so we determined that DEG6 was a target of GmWRKY46 in response to Pi starvation. Bioinformatics analysis showed that DEG6 was a eukaryotic aspartic protease family protein called AED1, which was part of a homeostatic feedback mechanism regulating systemic immunity (Breitenbach et al., 2014). In this study, we provided direct evidence of its involvement in nutrient stress. We speculated that there might be a similar homolog that would exist in soybean and be regulated by *GmWRKY46*. However, some effort is needed to identify which of the many homologous genes in the database may play a similar role in responding to low Pi stress. Meanwhile, in order to better demonstrate *GmWRKY46* enhanced Pi starvation tolerance via regulating *AtAED1*, further research is needed to determine if overexpressing *GmWRKY46* in the mutant of *ataed1* will no longer lead to enhancement of Pi starvation tolerance.

In addition, *AtAED1* is induced by salicylic acid (SA) (Breitenbach et al., 2014), and our RNA-seq results also indicated that the genes involved in SA synthesis and transport are affected. Whether *AtAED1* affects the tolerance to low Pi by affecting SA is an interesting research direction. After all, some research has shown that SA played a role in the P uptake and root development of plants (Khorassani et al., 2011; Wang et al., 2020). Furthermore, we did not find the reason why overexpression of *GmWRKY46* in transgenic plants promoted root development in this study, which is an urgent problem to be solved in the future.

## Conclusion

To conclude, we identified a WRKY TFs family gene from soybean, *GmWRKY46*, induced by Pi starvation. Overexpression of *GmWRKY46* enhanced tolerance to Pi starvation and affected root growth in transgenic plants. Also, *GmWRKY46* could enhance low Pi tolerance by activating *ATAED1*, a eukaryotic aspartyl protease family protein gene in transgenic *Arabidopsis*. Further studies should focus on the roles of *GmWRKY46* in soybean plants and find some similar *AtAED1* genes in soybean and their potential manipulation to improve Pi starvation tolerance or other agronomic traits.

## Data Availability Statement

The datasets presented in this study can be found in online repositories. The names of the repository/repositories and accession numbers can be found below: https://www.ncbi.nlm.nih.gov/bioproject/PRJNA724748.

## Author Contributions

CL and SY planned the research. CL, KL, XL, HR, MZ, and ZY performed the experiments. CL analyzed the data and wrote the manuscript. SY revised the manuscript. JG reviewed the manuscript. All authors approved the final manuscript.

## Funding

This work was supported by the National Transgene Science and Technology Major Program of China (2016ZX08004-005), the Fundamental Research Funds for the Central Universities (KYT201801), and the Program for Changjiang Scholars and Innovative Research Team in University (PCSIRT_17R55).

## Conflict of Interest

The authors declare that the research was conducted in the absence of any commercial or financial relationships that could be construed as a potential conflict of interest.

## Publisher's Note

All claims expressed in this article are solely those of the authors and do not necessarily represent those of their affiliated organizations, or those of the publisher, the editors and the reviewers. Any product that may be evaluated in this article, or claim that may be made by its manufacturer, is not guaranteed or endorsed by the publisher.
