# Supplementary material for: Transcription Factor GmWRKY46 Enhanced Phosphate Starvation Tolerance and Root Development in Transgenic Plants
Source: Front Plant Sci. 2021 Sep 14;12:700651. doi: 10.3389/fpls.2021.700651 (PMC8477037; doi:10.3389/fpls.2021.700651)
Supplement: Supplementary file 1 [file Data_Sheet_1.PDF]

Figure S1

(a)

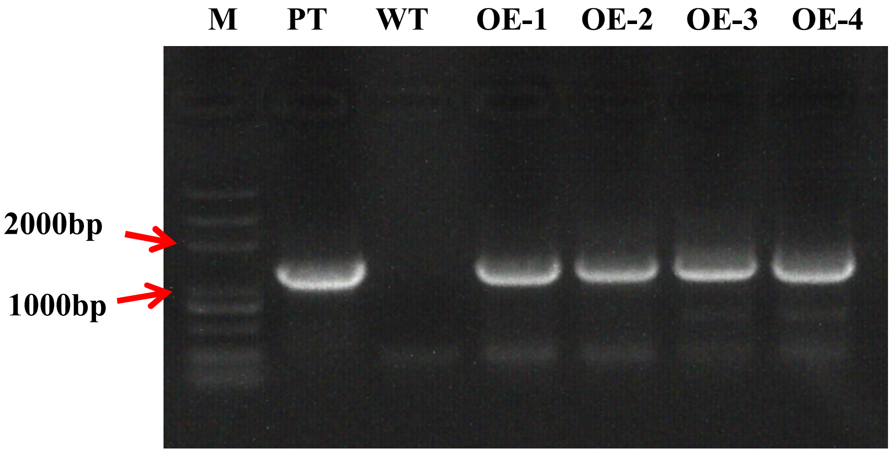

(b)

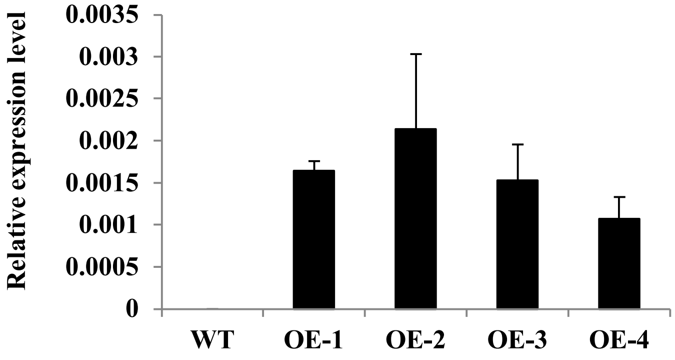

Figure S1. The *GmWRKY46*-overexpressing transgenic *Arabidopsis* was identified by PCR (a) and RT-qPCR (b). (a) *GmWRKY46* gene has a size of 1080bp fragment**.** M, DNA Marker DL 5000; WT, wild type *Arabidopsis* lines; PT, the binary vector pCAMBIA3301-*GmWRKY46*; OE-1 to 4, independent transgenic *Arabidopsis* lines. (b) Expression levels of *GmWRKY46* in the WT and four independent transgenic *Arabidopsis* lines (OE-1 to 4). Each bar is the meaning of three replicates with the standard deviation.

Figure S2

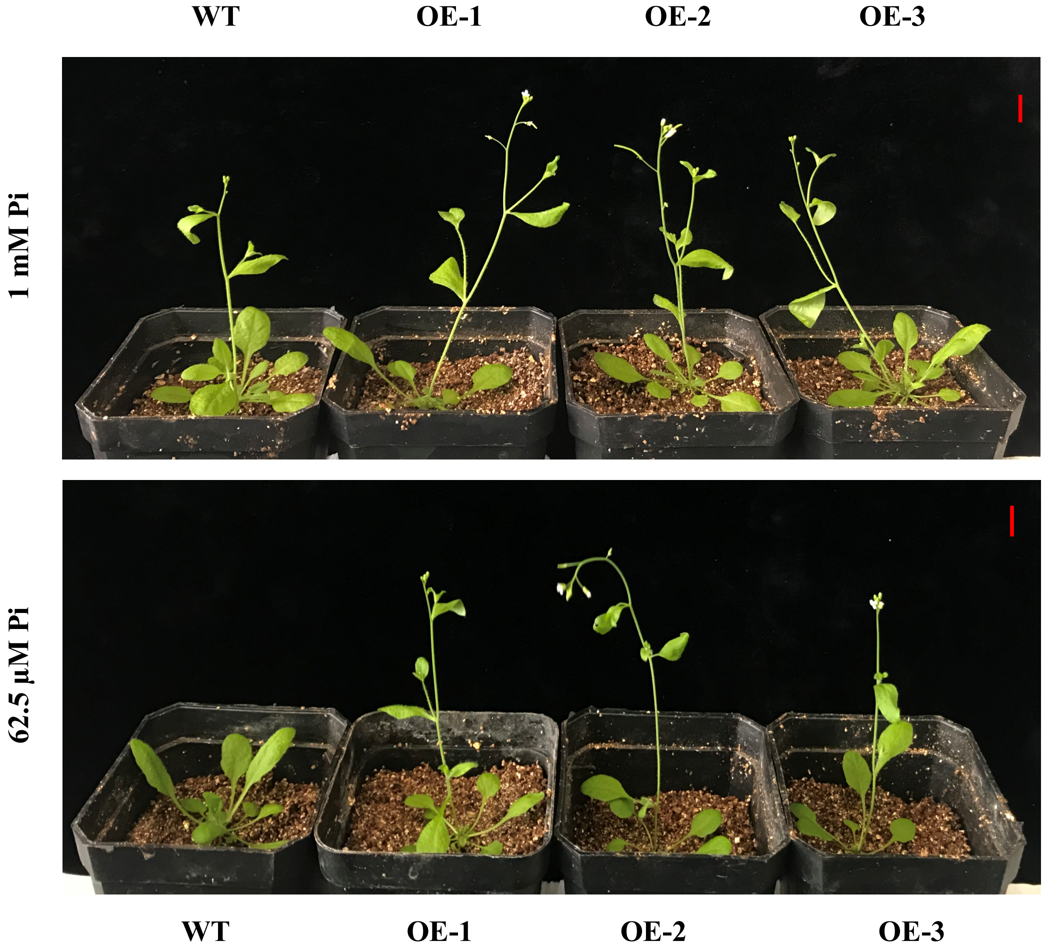

Figure S2. Overexpression of *GmWRKY46* enhanced tolerance to Pi starvation in transgenic *Arabidopsis*. Two-week-old seedlings were grown in the greenhouse for 20 d under 1 mM Pi and 62.5 µM Pi conditions. WT, wild type *Arabidopsis*, OE-1 to 3, independent transgenic *Arabidopsis* lines. Bars: 1 cm.

Figure. S3

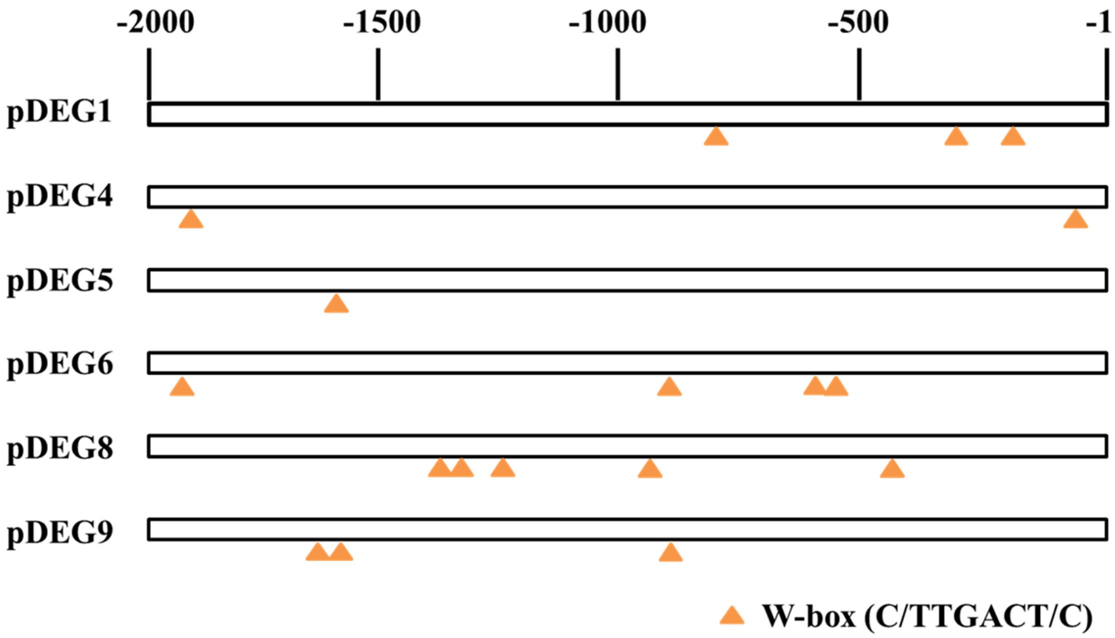

Figure. S3. W-box motif (C/TTGACT/C) analysis on promoter of 6 DEGs (DEG1, DEG4, DEG5, DEG6, DEG8 and DEG9). Promoter is a 2-kb sequence upstream of each gene start codon. Yellow triangle represents the position of W-box on the promoter.

Figure S4

(a)

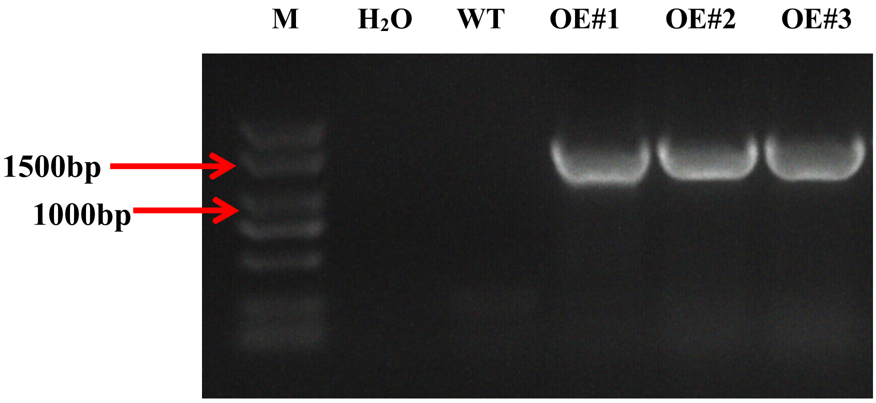

(b)

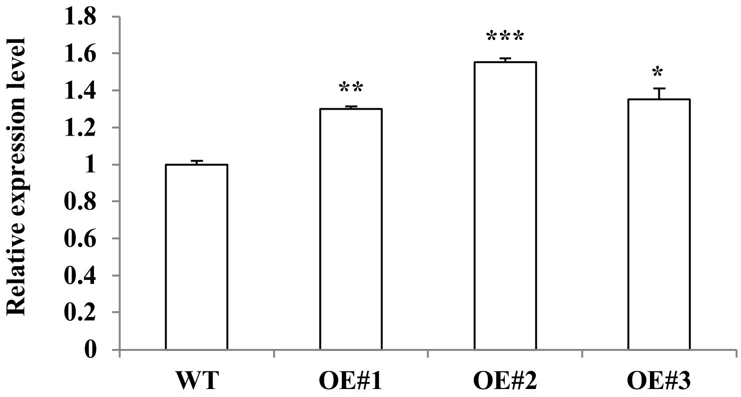

Figure S4. The *DEG6*-overexpressing transgenic *Arabidopsis* was identified by PCR (a) and RT-qPCR (b). (a) *DEG6* has a size of 1395bp fragment**.** M, DNA Marker DL 2000; H_2O, Distilled water; WT, wild type *Arabidopsis* lines; OE#1 to 3, independent transgenic *Arabidopsis* lines. (b) Expression levels of *DEG6* in the WT and three independent transgenic *Arabidopsis* lines (OE#1 to 3). Each bar is the meaning of three replicates with the standard deviation. Asterisks indicate significant differences between OE and WT (Student's *t*-test, *$P < 0.05$; **$P < 0.01$; ***$P < 0.001$)

Figure. S5

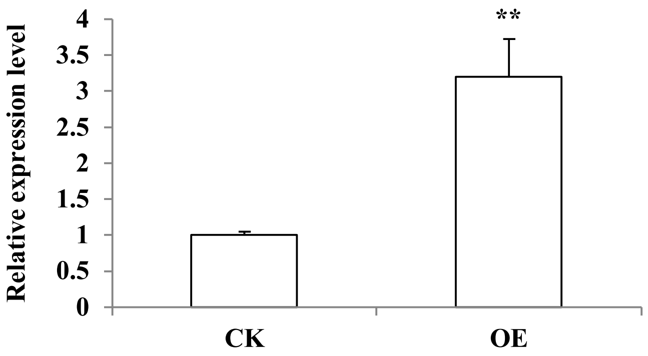

Figure. S5. Expression levels of *GmWRKY46* in transgenic hairy roots of soybean composite plants. CK represents soybean hairy roots transformed with the empty vector; OE means transgenic soybean hairy roots with overexpressing *GmWRKY46*. Each bar is the mean of three replicates with the standard error. Asterisk indicated a significant difference between OE and CK (Student's *t*-test, $^{**}P < 0.01$).
