# Supplementary material for: Transcription Factor GmWRKY46 Enhanced Phosphate Starvation Tolerance and Root Development in Transgenic Plants
Source: Front Plant Sci. 2021 Sep 14;12:700651. doi: 10.3389/fpls.2021.700651 (PMC8477037; doi:10.3389/fpls.2021.700651)
Supplement: Supplementary file 2 [file Data_Sheet_2.PDF]

Table S1. Sequences of primers used in this study for vector construction

Primer name	Forward primer (5'-3')	Reverse primer (5'-3')
pJIT166-GmWRKY46-GFP	CTCTAGAATGGAGAG<br>TGACTTGAGCTGGG	CGGATCCGCACAA<br>AAATCCTGGGGTATTG
pCAMBIA3301-GmWRKY46-GFP	AACACGGGGGACTCT<br>TGACAATGGAGAGTG<br>ACTTGAGCTGGG	GCCCTTGCTCACCATA<br>GATCTGCACAAAAATC<br>CTGGGGTATTG
pAbAi-F1	AGCTTCAATCCTGAC<br>TTGGAACAATCCTGA<br>CTTGGAACAATCCTG<br>ACTTGGAAC	TCGAGTTCCAAGTCAG<br>GATTGTTCCAAGTCAG<br>GATTGTTCCAAGTCAG<br>GATTGA
pAbAi-F2	AGCTTAGAGATTGAC<br>CGCGAGAGAGATTGA<br>CCGCGAGAGAGATTG<br>ACCGCGAGC	TCGAGCTCGCGGTCAA<br>TCTCTCTCGCGGTCAA<br>TCTCTCTCGCGGTCAA<br>TCTCTA
pAbAi-F3	AGCTTCGGATCTGAC<br>CGGCTTCGGATCTGA<br>CCGGCTTCGGATCTG<br>ACCGGCTTC	TCGAGAAGCCGGTCAG<br>ATCCGAAGCCGGTCAG<br>ATCCGAAGCCGGTCAG<br>ATCCGA
pAbAi-F4	AGCTTCGGCTTTGAC<br>TTGTTTCGGCTTTGA<br>CTTGTTTCGGCTTTG<br>ACTTGTTTC	TCGAGAAACAAGTCA<br>AAGCCGAAACAAGTC<br>AAAGCCGAAACAAGT<br>CAAAGCCGA
pGADT7-GmWRKY46	GTGGGCATCGATACG<br>GGATCCATGGAGAGT<br>GACTTGAGCTGGG	ACGATTCATCTGCAGC<br>TCGAGGCACAAAAATC<br>CTGGGGTATTG
pGADT7- GmWRKY46	ATGGCCATGGAGGCC<br>GAATTCATGGAGAGT<br>GACTTGAGCTGGG	CCGCTGCAGGTCGACG<br>GATCCGCACAAAAATC<br>CTGGGGTATTG

Table S2. Sequences of primers used in this study for RT-qPCR

Gene	Forward primer (5'-3')	Reverse primer (5'-3')
GmWRKY46	GGGATGGAGGTAGCAAGGAA	TGCAACTCGGAAGTTGATGC
DEG1	GCAGCAGTAAGGTGGTGATG	TGGTTGGAGTCGTTGGAGTT
DEG3	GTGGAGGAGTTACGGAGGAG	CACGCGCCTGATCTAAAGAC
DEG4	CAGCTCTCGAGGTTGCTAGA	AGCAAGCTGGAGCCAGAATA
DEG5	ATGGAGCCCTCACACTTTCA	GTCTTCACACCATGCTCGTC
DEG6	TTCACTGGCTTGGATACGGT	TTGATCGGCAACGAAATCCC
DEG7	ACGTGTCTTCGATCTAGCGT	TTCTCTGGTCCTCCACGAAC
DEG8	CGAGCCAGAGGATCTCACAT	CGTGATCCGTCTGATATGCG
Actin	CCTCAACCCAAAGGTCAACAG	GACCAGCGAGATCCAAACGAA
Actin2/8	ACGGTAACATTGTGCTCAGTGGTG	CTTGGAGATCCACATCTGCTGGA

Table S3. Sequences of primers used in this study for ChIP-qPCR

Primer name	Forward primer (5'-3')	Reverse primer (5'-3')
ChIP-qPCR-F1	ATACGACATGTAGATTTGCTTTCTT	AGGTTACACTCAAGGAAAAAGATG
ChIP-qPCR-F2	GTGATGATCCTTACCTGGAATCT	GACTGGCTCAAATCAAGTACCA
ChIP-qPCR-F3	GGACAAGTAAGAATCGGAGCG	GGTCAGATCCGCTTCATCCTC
ChIP-qPCR-F4	GGCTTTGACTTGTTTTCTCCG	AAACTACAAAAGCCATTTCCTCC
Actin2	GTTAGCAACTGGGATGATATGG	CAGCACCAATCGTGATGACTTGCCC

Table S5. GO functions of 11 DEGs

DEG	ID	GO Function
DEG1	AT1G28330	GO:0003674//molecular_function
DEG2	AT3G01345	-
DEG3	AT3G49570	GO:0003674//molecular_function
DEG4	AT4G14365	GO:0046914//transition metal ion binding; GO:0043167//ion binding; GO:0046872//metal ion binding; GO:0043169//cation binding; GO:0008270//zinc ion binding; GO:0005488//binding
DEG5	AT5G10180	GO:0008509//anion transmembrane transporter activity; GO:0022892//substrate-specific transporter activity; GO:0022857//transmembrane transporter activity; GO:0015075//ion transmembrane transporter activity; GO:0015103//inorganic anion transmembrane transporter activity; GO:1901682//sulfur compound transmembrane transporter activity; GO:0005215//transporter activity; GO:0015116//sulfate transmembrane transporter activity; GO:0022891//substrate-specific transmembrane transporter activity
DEG6	AT5G10760	-
DEG7	AT5G26220	GO:0003674//molecular_function
DEG8	AT5G54610	-
DEG9	AT5G60720	GO:0003674//molecular_function
DEG10	AT5G65080	GO:0001071//nucleic acid binding transcription factor activity; GO:0003700//transcription factor activity, sequence-specific DNA binding
DEG-11	XLOC_025428	-
